# Cortical Electrophysiological Markers of Language Abilities in Children with Hearing Aids: A Pilot Study

**DOI:** 10.1155/2014/198153

**Published:** 2014-08-19

**Authors:** David Bakhos, Hélène Delage, John Galvin, Emmanuel Lescanne, Sylvie Roux, Frédérique Bonnet-Brilhault, Nicole Bruneau

**Affiliations:** ^1^CHRU de Tours, Service ORL et Chirurgie Cervico-Faciale, boulevard Tonnellé, 37044 Tours, France; ^2^INSERM U930, 37044 Tours, France; ^3^Université François-Rabelais de Tours, CHRU de Tours, UMR-S930, 37044 Tours, France; ^4^Laboratoire de Psycholinguistique Expérimentale, Faculté de Psychologie et des Sciences de l'Education, Université de Genève, 40 boulevard du pont d'Arve, 1211 Genève 4, Switzerland; ^5^Department of Head and Neck Surgery, David Geffen School of Medicine, UCLA, Los Angeles, CA 90095, USA; ^6^CHRU de Tours, Service de Pédopsychiatrie, boulevard Tonnellé, 37044 Tours, France

## Abstract

*Objective*. To investigate cortical auditory evoked potentials (CAEPs) in pediatric hearing aid (HA) users, with and without language impairment. *Design*. CAEPs were measured in 11 pediatric HA users (age: 8–12 years) with moderate bilateral sensorineural hearing loss (HL); participants were classified according to language ability. CAEPs were also measured for a control group of 11 age-matched, normal-hearing (NH) children. *Results*. HL children without language impairment exhibited normal CAEPs. HL children with language impairment exhibited atypical temporal CAEPs, characterized by the absence of N1c; frontocentral responses displayed normal age-related patterns. *Conclusion*. Results suggest that abnormal temporal brain function may underlie language impairment in pediatric HA users with moderate sensorineural HL.

## 1. Introduction

The human cochlea is mature at birth; however, axonal, dendritic, and synaptic maturation and myelination continue to develop in the brainstem into early childhood and in the cerebral cortex into late childhood [1]. Auditory development and speech perception are guided by relevant acoustic and linguistic information experienced early in life to assure cortical maturation [2]. Hearing loss (HL) can be deleterious to children's speech and language development due to reduced quality and quantity of auditory input, and these developmental difficulties can have a cascading effect on social, academic, and (later) occupational success [3]. Given the restricted auditory input, abnormal cortical auditory maturation can occur in children with sensorineural HL, suggesting that the root cause may lie in the inner ear [4].

In case of moderate HL in childhood, hearing aids (HAs) can improve speech audibility and facilitate language development, assuming that the auditory cortical areas are functional [5]. However, individual variation in language performance has been observed in children with mild to moderate sensorineural HL [6]. Approximately 50% of children fitted with HAs for moderate HL have language impairment despite normal aided audiometric thresholds [7, 8]; the same finding was observed in adolescents [9]. Other studies have suggested that impairment in basic auditory processing might contribute to language difficulties in children with specific language impairment [10–12]. Language impairment might therefore be related to abnormal cortical auditory processing, which can be investigated using cortical auditory evoked potentials (CAEPs).

The successive peaks of CAEPs correspond to the spatiotemporal involvement of the cortical auditory generators and are therefore influenced by cortical maturation [13–15]. Whereas the morphology of frontocentral CAEPs is strongly influenced by age and provides an important index of auditory function and plasticity, the morphology of temporal CAEPs remains stable throughout childhood [16], with the successive negative N1a and N1c peaks occurring at approximately 80 and 160 ms, respectively [17]. These temporal responses (T-complex) represent the activity of the secondary auditory cortex [17]. The frontocentral CAEPs of children mainly exhibit two successive positive-negative peaks (P100 and N250) occurring at approximately 100 and 200 ms, respectively. At 8–11 years, adult-like CAEP waveforms progressively emerge, with the successive N1b, P2, and N250 peaks occurring at approximately 100, 180, and 220 ms, respectively. Given that speech information is transmitted at somewhat rapid rates (100 to 200 words per minute) [18], short interstimulus intervals (<750 ms) have sometimes been used to study the effect of the stimulus rate on frontocentral CAEPs in children that were categorized according to age [13]. Indeed, stimulus rate has been recognized as a marker of cortical auditory maturation [13]. Only one study has examined the influence of stimulus rate on temporal responses in children [19]. In that study, the amplitude of the temporal negative peak corresponding to Tb increased with interstimulus interval (350, 700, 1400, and 2000 ms), indicating a long refractory period for the underlying generator. Thus, it appears that long interstimulus intervals may be favorable for identifying successive peaks of the T-complex, and their asymmetry.

The aim of this study was to use CAEPs to investigate cortical auditory processing in regard to pediatric HA users with different levels of language ability. We hypothesized that temporal auditory responses and/or their sensitivity to stimulus rate would reflect different levels of language ability in these patients.

## 2. Patients and Method

### 2.1. Subjects

In this cortical electrophysiological study, we included children with symmetrical bilateral sensorineural HL fitted with HAs and aged between 8 and 12 years. Children in this age range were chosen for this pilot study as they are likely to understand and follow instructions during testing (i.e., CAEP, audiometry, and language tests). Participants were recruited from the Pediatric Unit of the Otolaryngology Department during clinical follow-up visits.

We reviewed patient charts of pediatric HA users. Thirty children had symmetrical bilateral sensorineural HL and were between 8 and 12 years old. Eleven children (8 males, 3 females), aged between 8.3 and 12.8 years (mean: 10.9 yrs), fitted with bilateral HAs for a bilateral moderate sensorineural HL were accepted into the study. A control group of 11 age- and gender-matched NH children with normal language development, as evaluated using the battery of oral language (evaluated with BILO battery including receptive and expressive language skills; see below for explanation), was also recruited for the study. For all participants, French was the main language spoken at home. All participants were right-handed.

Aided and unaided pure tone average (PTA) thresholds (averaged across audiometric frequencies 0.5, 1, 2, and 4 kHz) were <20 dB for pediatric HA and NH participants, respectively. Demographic data for pediatric HA users are shown in Table 1. All participants used oral communication and were enrolled in mainstream schools.

The Ethics Committee of the University Hospital of Tours approved the protocol, and written informed consent was obtained from the parents and assent from the children.

Spoken language and literacy skills were assessed using BILO, a set of standardized, computerized French language tests [20]. The BILO battery was standardized over a population of 272 primary and middle school students. In the BILO battery, phonology is assessed by a word repetition task using 42 words of increasing length and/or complexity. Expressive vocabulary is assessed using a classical naming task. Expressive grammar is evaluated using a sentence completion task and assesses the ability to produce a variety of specific grammatical morphemes: nominal, adjectival, and verbal inflexion, irregular plurals, prepositions, passive structure, and pronominal clitics. Reading is assessed using a timed task in which the child has to read, in 60 seconds, as many words as possible from a list of words of increasing difficulty. Spelling is tested using a word identification task, in which the child is presented with words that are correctly spelled, contain homophonic or nonhomophonic misspellings, or merely belong to the same semantic field and must decide whether the word corresponding to the picture is correct. Lexical judgment is tested by asking the child to decide whether there is concordance between a word and picture presented simultaneously. Grammatical judgment is tested by asking the child to decide whether a sentence corresponding to a picture is grammatically correct.

As described in Delage and Tuller [9], BILO scores were converted to z-scores. Language impairment was defined as scores on two or more of the language subtests that were 1.2 standard deviations below the norm. Six of the pediatric HA participants were deemed as having “good” language ability (HL+), with BILO > 1.2, while five were deemed as having “fair” language ability (HL−), with BILO < 1.2 (see Table 1). They were age-matched to control group of 11 NH children categorized according to similar language ability (HL+ controls and HL− controls). Six children (4 males and 2 females) were included in the HL+ controls (mean age: 11.6 years ± 0.7) and 5 children (4 males and one female) were included in the HL− controls (mean age: 9.4 years ± 1.9).

### 2.2. CAEPs Assessments

#### 2.2.1. Stimuli and Procedure

Participants were tested while sitting on an armchair in a dimly lit, sound-insulated room; pediatric HA participants were tested while wearing their HAs. Participants' mother or father accompanied them in the room during testing. The stimuli were comprised of 50-ms tone bursts (1100 Hz) delivered through two loudspeakers placed symmetrically on each side of the computer screen. The tone stimuli were presented via Neuroscan Stim^2^ software. The stimuli were presented at four different interstimulus intervals: 700 (i1), 1100 (i2), 1500 (i3), and 3000 (i4) ms. The sound intensity was 70 dBA measured at the head of the participant.

#### 2.2.2. Electroencephalogram (EEG) Recording

EEG recordings were obtained using 28 Ag-AgCl cup electrodes (Ffz, Fz, Cz, Pz, O1, F3, FC1, FT3, C3, T3, CP1, TP3, P3, T5, and F7) and their counterparts on the right hemiscalp. Electrodes were placed according to the 10-20 system, as well as the left and right mastoids (M1 and M2), and referenced to the nose. In addition, to detect ocular artifacts, vertical electrooculogram (EOG) data were recorded from two electrodes above and below the right eye (vertical bipolar).

The EEG and EOG were digitized (Neuroscan Synamps amplifier, Scan 4.3, Compumedics Corp., El Paso, TX) at a sampling rate of 500 Hz. The EEG was amplified and bandpass-filtered (0.3–100 Hz). Electrode impedances were kept below 10 kΩ. Eye movement artifacts were eliminated using a spatial filter transform developed by Neuroscan, and EEG periods with movement artifacts were rejected manually. A digital zero-phase-shift low-pass filter (30 Hz) was then applied to the EEG.

#### 2.2.3. Data Analysis

CAEPs were analyzed with the ELAN software [21]. Analysis was performed for waveform peaks occurring at frontocentral sites (N1b-P2-N250) and at temporal sites: N1a, N1c, P1t (the positive peak between these N1a and N1c), and P2t (the positive peak following N1c).

The influence of interstimulus interval on each peak of the CAEPs was analyzed using Friedman nonparametric analyses of variance. Amplitudes and latency peaks measured in HL+ and HL− children and the control group were compared using nonparametric Mann-Whitney rank tests.

## 3. Results

HL+ and HL− children did not differ, using Wilcoxon Mann-Whitney test, on age at testing (HL+: 11.7 years old ± 0.8; HL−: 9.5 years old ± 1.7; *P* = 0.08) or experience with hearing aids (HL+: 6.4 years, HL−: 5.7 years; *P* = 0.4).

### 3.1. Frontocentral Responses

**Figure 1:**
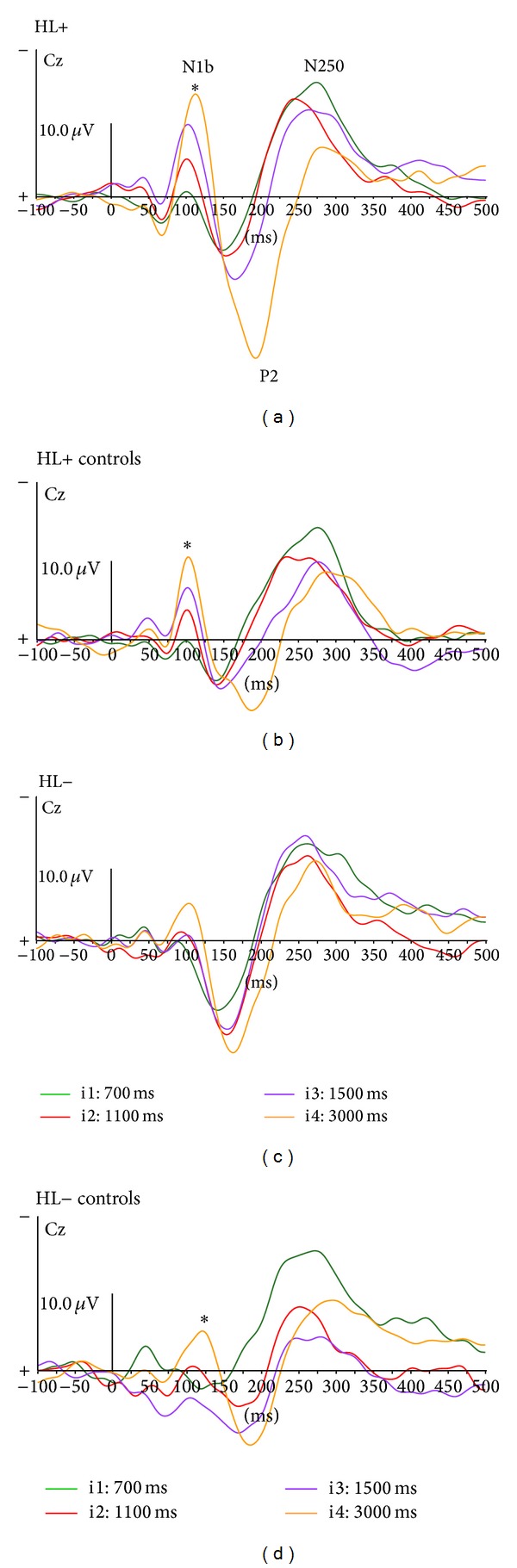
Cortical AEPs. Midline responses (Cz) at different interstimulus intervals (i1, i2, i3, and i4) for the HL+ (a), HL+ controls (b), HL− (c), and HL− controls (d). The asterisks indicate significant effect of interstimulus interval (*P* < 0.05).

Frontocentral CAEPs displayed similar successive N1b-P2-N250 peaks across the four groups. No significant difference was observed for the N1b peak amplitudes and latencies between HL+ and HL+ controls, or between HL− and HL− controls (Figure 1). The N1b peak amplitude was smaller for HL− than for HL+ participants and for HL− controls than for HL+ controls. This might be related to age differences, as the HL− and HL− control participants were younger (mean age was, resp., 9.5 and 9.4 years) than the HL+ and HL+ control participants (mean age was, resp., 11.7 and 11.6 years). A significant effect of interstimulus interval was observed on N1b amplitude for all groups except for the HL−. The N1b peak increased with interstimulus interval in HL+ (*P* = 0.01), HL+ controls (*P* = 0.03), and HL− controls (*P* = 0.04). There was a greater P2 peak amplitude in HL+ than in HL+ controls and was significant at i3 (*P* = 0.02) and at i4 (*P* = 0.004). The P2 peak was better individualized in HL− children than in matched controls. The N250 peak amplitude and latency did not vary with interstimulus interval and no significant differences across groups were observed.

### 3.2. Temporal Responses

Because no significant effect of interstimulus interval was found for the amplitude and latency of the successive peaks recorded at temporal sites N1a, P1t, N1c, and P2t, the CAEPs were averaged across the 4 interstimulus intervals to increase the signal to noise ratio (SNR).

**Figure 2:**
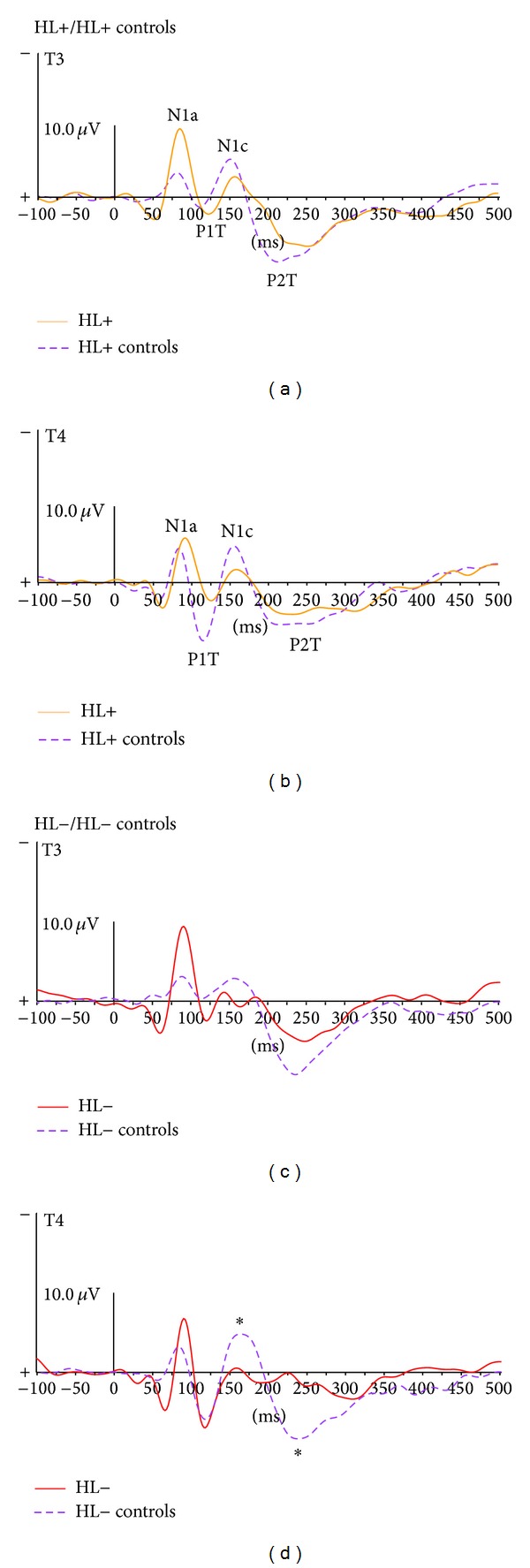
CAEP T3 (a and c) and T4 temporal responses (b and d) at i1, i2, i3, and i4 for the HL+ and HL+ control groups (a and b) and for the HL− and HL− control groups (c and d). The asterisks indicate significant differences (*P* < 0.05) for the peak amplitude wave.

Although the grand average N1a peak amplitude was greater in the HL+ and HL− groups than in their respective controls (mainly on the left temporal site), the difference was not statistically significant. The subsequent P1t, N1c, and P2t waves also were not significantly different between the HL+ and HL+ control groups. However, the N1c and P2t amplitudes were significantly smaller (only on the right temporal site) in the HL− group than in the HL− controls (N1c: HL− = −0.8 *μ*V ± 1.3, HL− controls = −3.1 *μ*V ± 0.9, U = 2, *P* = 0.03; P2t: HL− = 3.1 *μ*V ± 0.6, HL+ controls = 5.4 *μ*V ± 1.4, U = 2, *P* = 0.03). This difference between the HL− group and the HL− controls was not observed for either latency or amplitude of the preceding P1t (Figure 2).

## 4. Discussion

The present pilot study provided interesting preliminary findings regarding the relationship between CAEP characteristics and language ability in 8- to 12-year-old pediatric HA users. Atypical CAEPs were observed at temporal recording sites for pediatric HA users with some degree of language impairment. In normal development, temporal CAEPs typically display a stable morphology through childhood and particularly for the age range of the present study, with successive N1a and N1c peaks [16, 17], suggesting that cortical auditory maturation does not change the morphology of temporal responses for children between 8 and 12 years old.

In our study, despite a normal (or greater than normal) amplitude of the early temporal peaks (N1a, P1t) for all pediatric HA patients, the later temporal responses (N1c and P2t) were reduced or absent in the HL− group. This does not appear to be due to an absence of cortical auditory input because waves N1a and P1t were present and normal in all the pediatric HA patients. N1c wave was absent or smaller in the HL− group. This relationship between N1c abnormalities (N1c being reduced or absent) and language impairment has previously been shown in other clinical populations with language impairment such as in children with autism [22], Down's syndrome [23], or specific language impairment [24]. Because generators of N1c are located at the lateral part of the superior temporal gyrus [25–27], the present results emphasize the importance of these cortical areas in language processing.

Relatively few studies have investigated CAEP temporal responses in pediatric HA users. Most of these studies have focused on frontocentral responses (especially the latency of the P1 peak recorded at the vertex) in deaf children who use cochlear implants [28–30]. These studies found that P1 peak latency can be a biomarker of auditory cortex maturation in children with congenital hearing loss and that this P1 peak latency will decrease with auditory rehabilitation. In the present study, the P1 peak latency did not differ between HA patients and NH controls, suggesting that the HA provided enough auditory input to allow maturation of the primary auditory cortex as found in cochlear-implanted children.

Unlike the temporal responses, the frontocentral responses of normally developing children are greatly influenced by age. The smaller N1b peak amplitude in the HL− and HL− control groups, compared to the well-defined peaks and greater amplitude of N1b in the HL+ and HL+ control groups, might be due to age differences between groups. This result is in agreement with the literature indicating the emergence of N1b at approximately 8–10 years of age and greater N1b amplitude at 10–12 years of age. The greater P2 peak amplitude observed in HL+ and HL− groups than in the controls may be related to HA amplification, as described in previous studies [31, 32]. N1b peak amplitude increased with interstimulus interval in the HL+ group, similar to the HL+ controls and previous studies [5]; the effect of interstimulus interval was not observed for the HL− group. This finding might be related to language impairment or the younger age of the HL− group, as a significant effect of interstimulus interval was observed for the HL− controls.

In this study, children with HA and good language ability (HL+) were older than those with language impairment (HL−). Language abilities were evaluated with BILO, which is calibrated in order to allow comparisons in language ability across age groups. Moreover, CAEPs were compared with age- and gender-matched control children.

Although further longitudinal studies are needed with larger sample of children, these preliminary results suggest that abnormal CAEP responses recorded at temporal sites might underlie language impairment in pediatric HA users who have a moderate sensorineural HL.

## Figures and Tables

**Table 1:** Demographic information for the pediatric HA users with bilateral moderate sensorineural HL.

Subject	Gender	Age test (yrs; months)	Duration of auditory deprivation (months)	Age at which child received HA (yrs; months)	Experience xith HA (yrs; months)	Aetiology	Right PTA	Left PTA	BILO score	Group
1	M	8; 3	6	3; 5	4; 10	Unknown	63	69	−1.34	HL−
2	M	8; 5	3	2; 1	6; 4	Familial	64	65	−3.53	HL−
3	M	8; 11	4	2	6; 11	Familial	66	66	−1.46	HL−
4	F	9; 7	2	6; 1	3; 6	Familial	56	56	−5.31	HL−
5	M	10; 5	2	5; 11	4; 6	Unknown	45	41	−0.97	HL+
6	M	11; 5	2	5; 11	5; 6	Unknown	41	43	−0.2	HL+
7	M	11; 8	1	3; 5	8; 3	Familial	55	66	−0.46	HL+
8	F	12; 1	18	8; 11	3; 2	Unknown	40	41	−0.16	HL+
9	F	12; 2	6	4; 10	7; 4	Familial	59	45	1.31	HL+
10	M	12; 6	5	8; 5	4; 1	Familial	41	41	−1.25	HL−
11	M	12; 10	6	5; 2	7; 8	Unknown	41	42	−0.5	HL+

Note: M: male; F: female; yrs: years; HA: hearing aid; PTA: aided pure tone audiometric threshold averaged over 0.5, 1, 2, and 4 kHz; BILO: Batterie Informatisée du Langage Oral; HL+: good language ability; HL−: fair language ability.
